# ﻿A new species of *Veronica* (Plantaginaceae) from Western Iran

**DOI:** 10.3897/phytokeys.237.115003

**Published:** 2024-01-29

**Authors:** Mahfouz Advay, Dirk C. Albach, Moslem Doostmohammadi

**Affiliations:** 1 Central herbarium, Scholar biology, College of Science of Tehran University, Tehran, Iran College of Science of Tehran University Tehran Iran; 2 Institut für Biologie und Umweltwissenschaften, Carl von Ossietzky-Universität, 26111 Oldenburg, Germany Carl von Ossietzky-Universität Oldenburg Germany; 3 Department of Biology, Shahid Bahonar University of Kerman, Kerman, Iran Shahid Bahonar University of Kerman Kerman Iran

**Keywords:** Endemic, Hawraman, Kurdistan, Schahu, *Veronica* subg. *Pentasepalae*

## Abstract

A new species, *Veronicakurdistanica* (Plantaginaceae), is described and illustrated. It grows on limestone cliffs in mountainous alpine areas of western Iran (Kurdistan province). The new species belongs to the species group of *V.kurdica* and is considered to be closely related to *V.daranica*, *V.khorassanica* and *V.kurdica*, with which the new species is compared. Molecular phylogenetic analysis of nrDNA (ITS) region confirms this relationship. *Veronicakurdistanica* is distinguished from the mentioned species by its glandular indumentum, length and shape of leaves and bracts, number of flowers per raceme, length and width of calyx and corolla, and size of capsules and seeds.

## ﻿Introduction

*Veronica* L. is the largest genus within the family Plantaginaceae in its current circumscription. The genus has cosmopolitan distribution and includes ca. 450 species (Albach et al. 2004; Fischer 2004). Species of *Veronica* have high ecological diversity and they are found in different habitats that range from arid steppes to aquatic habitats, from the sea level to high alpine regions (Albach et al. 2008). Its centers of diversity are the Eastern Mediterranean and Irano-Turanian regions, as well as in New Zealand (Albach et al. 2004).

Our knowledge of the species diversity of the genus in Iran was summarized in the “Flora Iranica” by Fischer (1981). He recognized 56 species occurring in Iran. However, several new taxa were added afterwards (Saeidi-Mehrvarz et al. 2001; Saeidi-Mehrvarz and Assadi 2003; Saeidi-Mehrvarz 2005, 2011) and the genus includes 61 species with 18 species endemic to the country according to the latest “Flora of Iran” (Saeidi-Mehrvarz 2011). At least two species have since been added to the flora of Iran (Doostmohammadi et al. 2021, 2022). Among these, most species occur in the Zagros and Alborz (also Elburz) Mountains of Iran, which are important centers of diversity of *Veronica*.

Among the recent collections from the northern Zagros Mountains, we identified a small chasmophytic species of *Veronica* which resembles *V.daranica* from central Zagros. Further morphological comparisons and molecular investigations revealed that the new collection belongs to a yet unknown species, which is described below.

## ﻿Materials and methods

During a scientific field survey on the western slope of the Zagros Mountains in Western Iran, the first author collected in 2015 specimens of a *Veronica* from Schahu Mountain in the Hawraman region, Kurdistan province. These specimens were compared with diagnostic keys reported in Floras (Fischer 1981; Saeidi-Mehrvarz 2011) and recent new records and species (Saeidi-Mehrvarz et al. 2001; Saeidi-Mehrvarz and Assadi 2003; Saeidi-Mehrvarz 2005). After careful examination with the different floras, we inspected images of type specimens from various online herbaria (BM, K, MPU, P), as well as related taxa in TUH, TARI, HKS and IRAN (herbarium acronyms according to Thiers 2016 and continuously updated). Subsequently, we measured several quantitative and qualitative morphological key traits of our specimens and related species (Table 1).

**Table 1.** The morphological differences among *Veronicakurdistanica* and its related taxa.

Characters	*V.kurdistanica*	*V.daranica*	*V.khorassanica*	V.kurdicasubsp.kurdica	V.kurdicasubsp.filicaulis
Stem	4–8 cm	up to 5 cm	6–12 (–25) cm	(5–) 10–20 (–30) cm	5–15 cm
Stem indumentum	densely glandular	glabrous	dense eglandular cinereous- subcrispate	eglandular velvety or rarely glabrous	eglandular pubescent, often glabrous
Leaf	4–10 mm long, 1–2 mm wide, oblong-obovate, elliptic to spathulate	2.5–5 mm long, 1–2 mm wide, narrowly elliptic, elliptic to spathulate	4–11 (–15) mm long, 0.8–1.5 mm wide, linear	5–10 (–16) mm long, 1–5 (–7) mm wide, linear above to elliptic-ovate below	2–6 (–8) mm long, 0.7–3 (–4.5) mm wide, linear to elliptic-ovate
Leaf indumentum	crispulate	glabrous	eglandular cinereous-crispate	eglandular or glabrous	eglandular or glabrous
Inflorescence	terminal, raceme 4–14 flowered	terminal, raceme 4– l2 flowered	axillary, raceme 15–20 (–60) flowered	axillary, raceme 5–**2**0 (–25) flowered	Axillary, raceme 5–15 (–20) flowered
Bract length and shape	2–2.5 mm, lanceolate	1.5–2 mm, spathulate	1–1.5 (–2.5) mm, linear, oblong-subspathulate	(1.5–) 2–3 (– 4) mm, oblong to obovate	1.5–3 mm, oblong
Pedicels length	1–1.5 mm long at anthesis, l.5–2 mm long in fruit	1–1.5 mm long at anthesis, l.5–2 mm long in fruit	2–3 (–4) mm long at anthesis and in fruit	1.5–6 mm at anthesis, 4–8 (–10) mm in fruit	0.5–3 mm at anthesis, 1.5–4 (–6) mm in fruit
Calyx	1.7–2 mm long and 1 mm wide at anthesis, 2–2.5 mm long and 1–1.2 mm wide in fruit	l .3– l .8 mm long and 0.4–0.7 mm wide at anthesis, 1.6–2.5 mm long and 0.5–1.2 mm wide in fruit	2–2.5 mm long at anthesis, 2.5–3 (–4) mm in fruit	1.5–3 mm at anthesis, 2–3 (–4) mm in fruit	1.5–3 mm at anthesis, 2–3 (–4) mm in fruit
Corolla color and size	purple, 2.5–3 mm long, 5 mm in diameter	purple, 2.3–2.5 mm long, 4–6 mm in diameter	purple, 6 mm in diameter	dark blue to violet blue, 4–6 mm long, 8–10 (–11) mm in diameter	Pink or purple or violet purple, 3–4 mm long, (5–)6–8 mm in diameter
Capsule	1.2–2.2 mm long and 2–2.1 mm wide	1.8–2.5 mm long and 2–2.5 mm wide	2.5–3.5 mm long, 3–3.5 mm wide	2.5–4 mm long, 4–5 mm wide	2–3 mm long, 3–4.5 mm wide
Seed	0.8–1.2 mm long, 0.3–0.5 mm wide	0.7–0.9 mm long, 0.5–0.7 mm wide	1.5–2.5 mm long, 1–1.5 mm wide	1.4–1.8 mm long, 1.1–1.4 (–1.6) mm wide	1.4–1.8 mm long, 1.1–1.4 (–1.6) mm wide

In order to determine the phylogenetic position of the new species, a phylogenetic analysis was conducted based on the nuclear ITS region. One new sequence of the ITS region of the herbarium specimen of the holotype (NO. 12808 HKS) was generated using ITS1 and ITS4 primers (White et al. 1990). The new sequence was added to a sequence matrix, based on published sequences of Doostmohammadi et al. (2022). All sequences were aligned using MAFFT v. 6.0 (Katoh and Toh 2010) and edited manually using PhyDE v. 0.9971 (Müller et al. 2010). Insertions and deletions (indels) were coded as binary characters using the simple indel coding approach, as implemented in SeqState v. 1.4.1 (Müller 2005). Bayesian inference (BI) was conducted using MrBayes v.3.2.6 (Ronquist et al. 2012) under GTR+Γ+I nucleotide substitution model. Two parallel runs of four MCMC chains including three heated and one cold chain were run simultaneously for four million generations, sampling every 200 generations. After removing 25% of the sampled trees as burn-in, a 50% majority-rule consensus tree was constructed.

## ﻿Results and discussion

The morphological and phylogenetic analyses indicate a close relationship between *Veronicakurdistanica* sp. nov., and *V.daranica* Saeidi & Ghahr., *V.khorassanica* Czernjak, and *V.kurdica* Benth. in DC. but ample morphological differences to suggest that *V.kurdistanica* is a distinct species.

### ﻿Taxonomy

#### ﻿ Veronica kurdistanica

Taxon classificationPlantaeLamialesPlantaginaceae

M.Advay sp. nov.

7EBB71DA-B2F9-5F76-85CC-9C1BF9166A01

urn:lsid:ipni.org:names:77335343-1

Fig. 1A–F ; Table 1

##### Type.

Iran – Kurdistan province, Kamyaran, Schahu mountain, 34°53'48"N, 46°33'43"E, 2700 m, 7 May 2015, *Advay 12808.* (holotype HKS! (Fig. 2), isotype TUH!).

**Figure 1.**
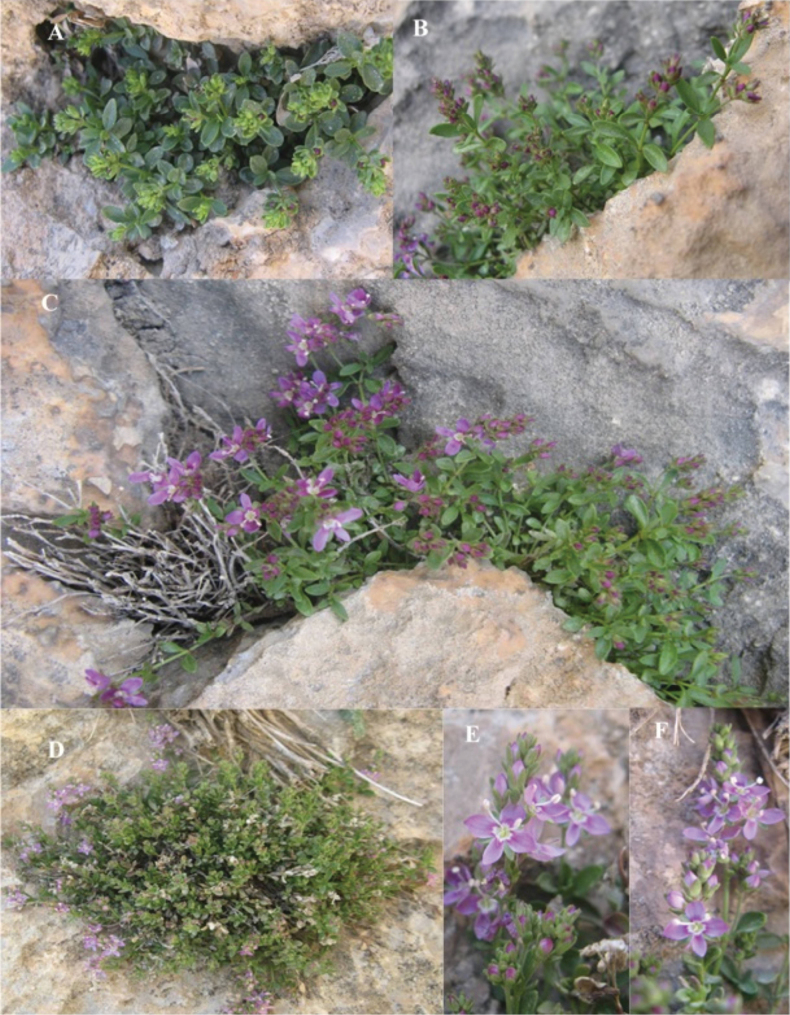
*Veronicakurdistanica***A–D** habitat and habit **E, F** corolla and inflorescence (photos by M. Advay).

**Figure 2.**
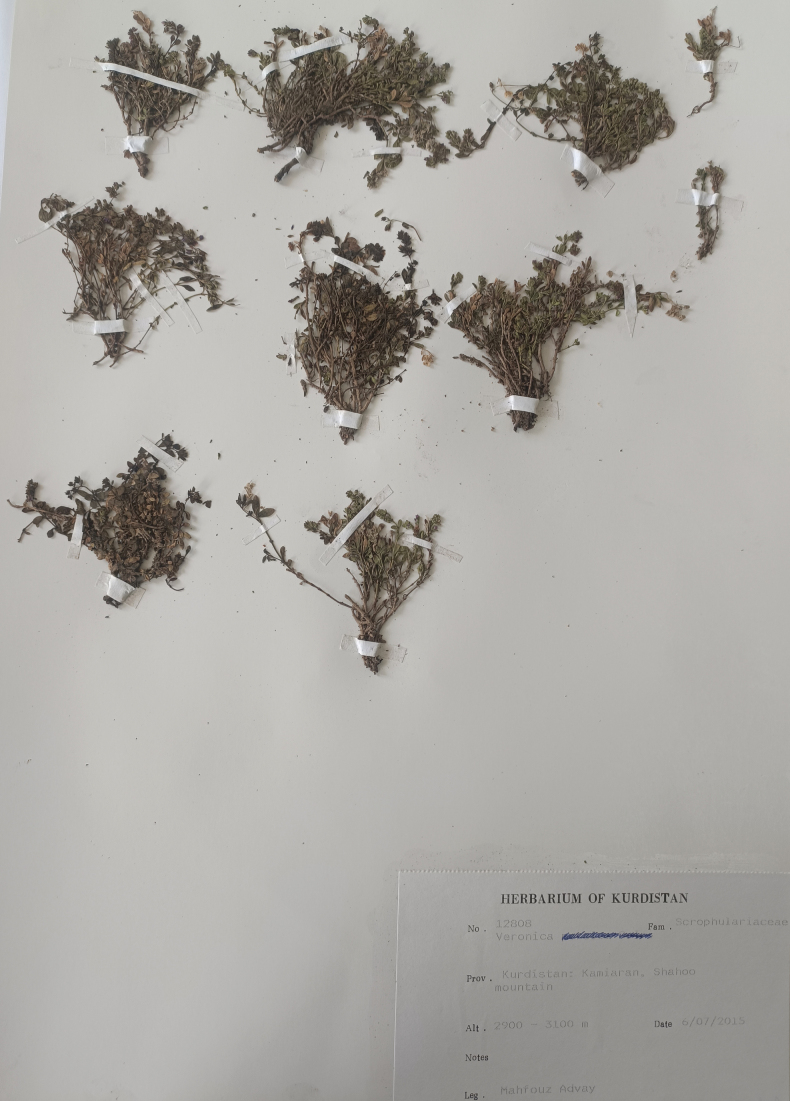
Holotype of *V.kurdistanica*.

##### Diagnosis.

**Figure 3.**
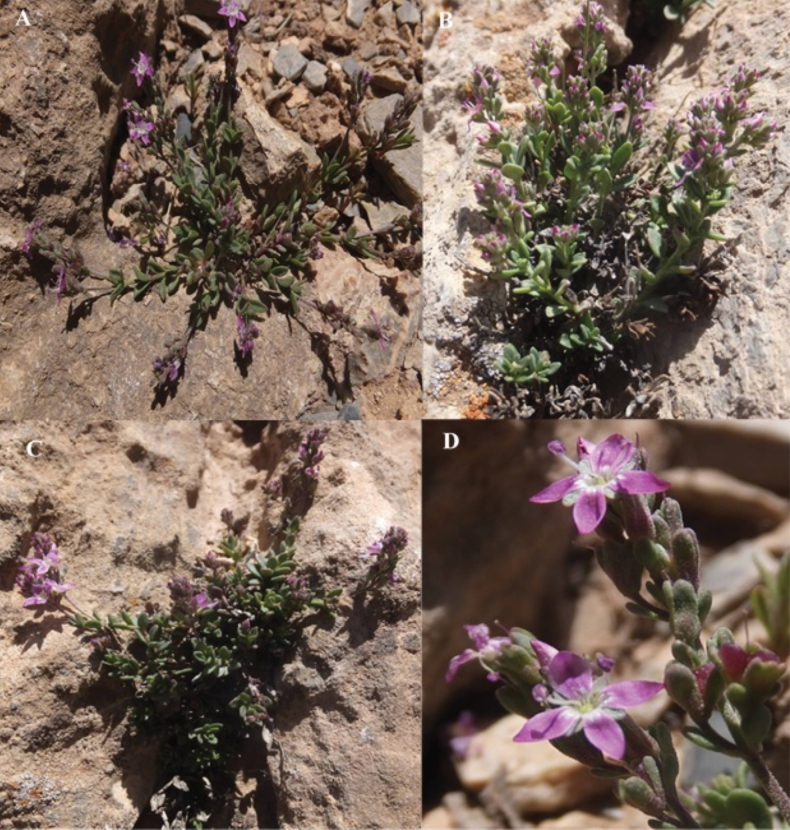
*Veronicadaranica***A–C** habitat and habit **D** corolla and inflorescence (photos by M. Doostmohammadi).

*Veronicakurdistanica* is similar to *V.daranica* (Fig. 3A–D) based on simple stems (branched only at the base), terminal racemes (rarely axillary), and purple petals. However, it is distinguished from *V.daranica* by the densely glandular (vs. glabrous) indumentum, obovate, elliptic to spathulate leaves that are 4–10 mm long (vs. narrowly elliptic, elliptic to spathulate and 2.5–5 mm long), lanceolate bracts that are 2–2.5 mm long (vs. spathulate and 1.5–2 mm long), capsule 1.2–2.2 mm long and 2–2.1 mm wide (vs. 1.8–2.5 mm long and 2–2.5 mm wide), and seeds 0.8–1.2 mm long and 0.3–0.5 mm wide (vs. 0.7–0.9 mm long, 0.5–0.7 mm wide) (Table 1). Also, *V.kurdistanica* is related to *V.khorassanica* but it is distinguished from the latter by the densely glandular indumentum (vs. dense eglandular subcrispate); oblong-obovate, elliptic to spathulate leaves (vs. linear), shorter, terminal racemes with 4–14 flowers (vs. axillary racemes with 15–20 (–60) flowers), longer bracts (2–2.5 mm) and calyces (1.7–2 mm long at anthesis, 2–2.5 mm long in fruit) (vs. bracts 1–1.5 (2.5) mm long, calyces 2–2.5 mm long at anthesis, 2.5–3 mm in fruit), and capsule (1.2–2.2 mm long and 2–2.1 mm wide vs. 2.5–3.5 mm long, 3–3.5 mm wide), and seed size (0.8–1.2 mm long and 0.3–0.5 mm wide vs. 1.5–2.5 mm long, 1–1.5 mm wide) (Table 1). Our new species also differs from *V.kurdica*, specifically by: stems 4–8 cm tall (vs. (5–)10–20(–30) cm), indumentum densely glandular (vs. eglandular or glabrous), raceme terminal and 4–14 flowered (vs. racemes axillary and 5–20 (–25) flowers), pedicels l.5–2 mm long in fruit (vs. 4–8 (–10) mm in fruit), corolla purple, 2.5–3 mm long, 5 mm in diameter (vs. dark to violet blue, 4–6 mm long, 8–10 (–11) mm in diameter) and capsule (1.2–2.2 mm long and 2–2.1 mm wide vs. 2–3.5 mm long, 3–5 mm wide) and seed size (0.8–1.2 mm long and 0.3–0.5 mm wide vs. 1.4–1.8 mm long, 1.1–1.4 (–1.6) mm wide) (Table 1).

##### Description.

Perennial plant, rhizome stout, plant 4–8 cm tall; stems woody, ascending at base, erect, ± densely glandular. Leaves 8–10 pairs, fleshy, lower leaves with petiole 8–10 mm long, 4–8 mm wide, gradually attenuate at base, oblong-obovate, elliptic to spathulate, cauline leaves 4–6 mm long, 1–2 mm wide, sessile above, entire, ± glandular, upper side more than lower side. Racemes 1–2, often terminal, 4–14 flowered, 0.5–1 cm long in flowering stage, elongated to 1–1.5 cm long in fruiting stage; hairs 0.3–0.5 mm long, glandular; bracts spathulate, 2–2.5 mm long, shorter than leaves, entire, densely glandular hairy on the upper surface, less so on the lower side; pedicel 1–1.5 mm long at anthesis, 1.5–2 mm long in fruit, pubescent with spreading yellowish glandular hairs. Calyx 1.5–2 mm long and 0.7–1 mm wide at anthesis, 2–2.5 mm long and 1–1.2 mm wide in fruit; lobes oblong, 0.4–0.8 mm long united at base. Corolla purple, 2.5–3 mm long, 4–5 mm in diameter, adaxial and lateral lobes elliptic, obtuse and abaxial lobe oblong, subacuminate, corolla tube white and internally densely pubescent; stamens with filaments 1.5–2 mm long, white; anthers ca. 1 mm, purplish to white; style 1.6–2.7 mm long, purplish, whitish at base. Capsule obcordate, 1.2–2.2 mm long and 2–2.1 mm wide, equaling or slightly overtopping the calyx, glandular hairs sparsely spreading. Seeds flat, oblong-elliptic, 0.8–1.2 mm long, 0.3–0.5 mm wide, brown-yellowish, surface reticulate.

##### Etymology.

Referring to Kurdistan Province (Western Iran), where the new species was discovered.

##### Distribution, habitat, and phenology.

**Figure 4.**
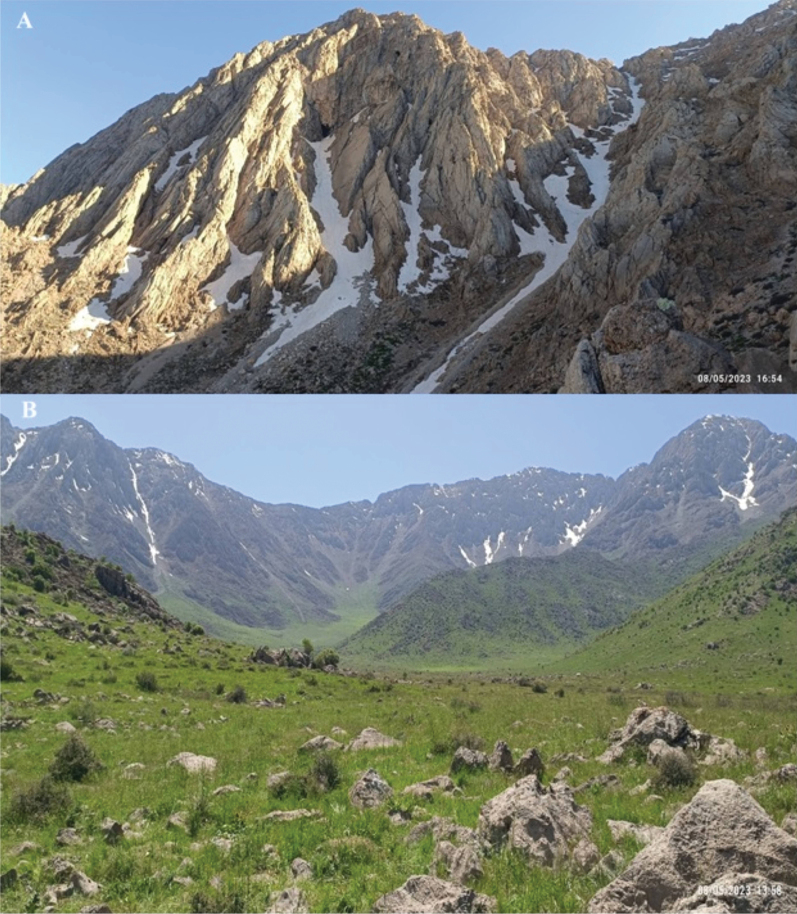
**A, B** habitat of *Veronicakurdistanica* (photos by M. Advay).

**Figure 5.**
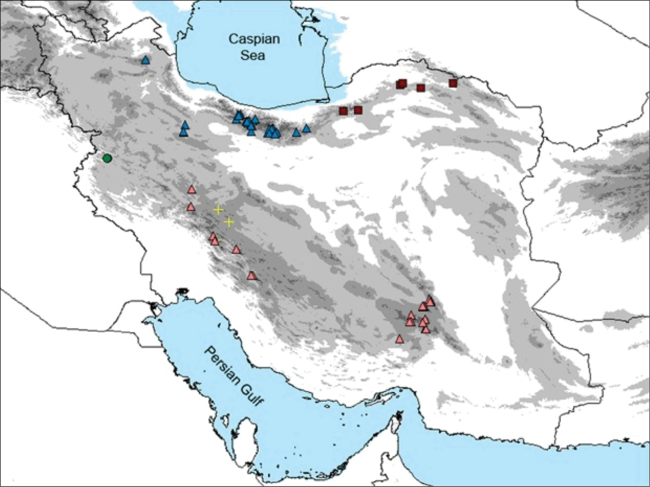
Distribution of *Veronicakurdistanica* (circle) and *V.daranica* (cross), *V.khorassanica* (square), V.kurdicasubsp.kurdica (blue triangle) and V.kurdicasubsp.filicaulis (pink triangle) in Iran.

The species is currently known from a few populations in Schahu mountain, Hawraman region, Kurdistan Province, western Iran. It is an Irano-Turanian phytogeographical element that grows on limestone cliffs of mountainous slopes of Schahu mountains, 2500–2950 m a. s. l. (Figs 4, 5). Flowering April to May, fruiting June.

##### Conservation status.

*Veronicakurdistanica* is observed in a restricted area of the Avroman (Hawraman) region in the province of Kurdistan. The estimated area of occupancy is less than 50 km^2^. The species is proposed to be classified as critically endangered (CR) following the IUCN criteria (2022).

##### Taxonomic notes.

**Figure 6.**
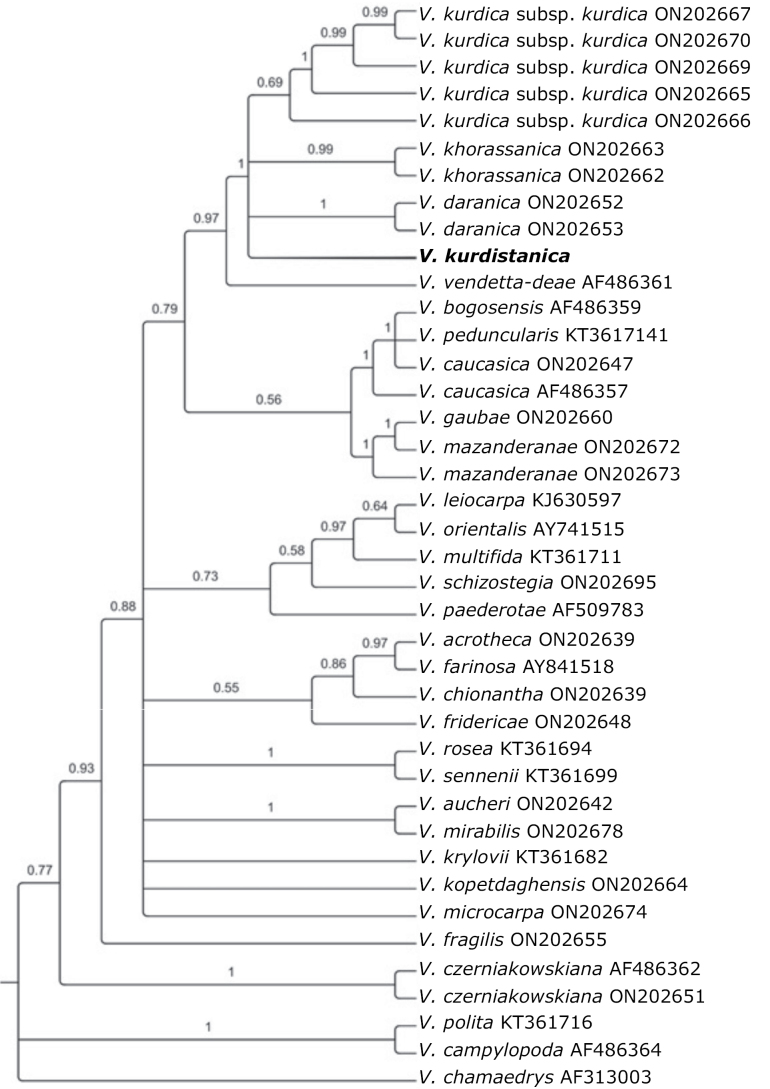
50% majority-rule consensus tree obtained from the Bayesian analysis of nrDNA ITS sequences. Posterior probabilities are given above the branches.

*Veronicakurdistanica* belongs to VeronicasubgenusPentasepalae (Benth.) M. M. Mart. Ort., Albach & M. A. Fisch. (Fig. 6), by far the most species-rich subgenus in Iran, with many perennial, mountainous species. It has been demonstrated that this subgenus has probably originated in the Iranian plateau, with several relict and morphologically isolated species distributed at present along the Zagros and Alborz Mountains (Doostmohammadi et al. 2022).

*Veronicakurdistanica* belongs to the *V.kurdica* species group and is morphologically closest to *V.daranica* (Fig. 3) but occurs more than 500 km apart (Fig. 5). The group has maximum support in the phylogenetic analysis (Fig. 6), although there is no morphological character setting this group apart from related species. Members of this group are all endemic, perennial species adapted to arid conditions; they are important constituents of the alpine flora of Iran. Species delimitation is sometimes difficult among the members of *V.kurdica* species complex. For instance, *V.daranica* differs from glabrous forms of V.kurdicasubsp.filicaulis (Freyn) M.A. Fisch. only by its dense, compact habit, thinner petals and some other subtle morphometric differences, but the molecular studies confirmed that it is a distinct species (Doostmohammadi et al. 2022). However, *V.kurdistanica* is a unique species within *V.kurdica* complex differing from all others by its glandular indumentum, apart from other subtler differences mentioned above. Other species are either glabrous or have an eglandular indumentum. It is not unusual in the genus to differentiate a species mainly on the basis of glandular indumentum and other subtle differences. An example is *V.porphyriana* Pavlov, which has often been included in *V.spicata* L. (*sensu lato*), but has been clearly differentiated based on DNA-based evidence (Khan et al. in press). In the molecular phylogenetic tree, our new species is assembled in a polytomy including *V.daranica*, *V.khorassanica*, and two subspecies of *V.kurdica* (Fig. 6). A comprehensive morpho-molecular study is required to delimitate further the species of this complex, both morphologically and geographically. The finding of this new species is noteworthy since it emphasizes that the Kurdistan region is an important center of diversity, despite being under-investigated. Thus, future field work may further increase the species number of the region.

##### Additional specimens

**(paratypes).** Iran – Kurdistan province, Kamyaran, Schahu mountain 34°53'48"N, 46°33'43"E, 2500 m, 10 May 2015, Advay 12829 (HKS), Kamyaran, Schahu mountain 34°54'30"N, 46°32'43"E, 2920 m, 15 May 2023, Advay 48735 (TUH).

## ﻿Conclusion

We here provide evidence for a new species from Iran, which belongs to the *V.kurdica* group of Veronicasubg.Pentasepalae. This finding emphasizes the need for further detailed floristic investigation of the region and further detailed phylogenetic investigations to find or clarify biogeographic patterns.

## Supplementary Material

XML Treatment for Veronica kurdistanica

## ﻿Additional information

### Author ORCIDs

Mahfouz Advay https://orcid.org/0000-0002-3542-532X

Dirk C. Albach https://orcid.org/0000-0001-9056-7382

Moslem Doostmohammadi https://orcid.org/0000-0003-1777-6676

### Data availability

All of the data that support the findings of this study are available in the main text. The new DNA sequence has been submitted to GenBank.
